# Focusing on the process: experiences and needs of Chinese post-esophagectomy patients and caregivers regarding home enteral nutrition

**DOI:** 10.3389/fnut.2025.1636203

**Published:** 2025-07-24

**Authors:** Huiyan Liao, Muxi Cheng, Yuxin He, Hui Li, Xinhui Song, Lei Zhong, Mei Li

**Affiliations:** ^1^Department of Thoracic Surgery, Nanfang Hospital, Southern Medical University, Guangzhou, Guangdong, China; ^2^School of Nursing, Southern Medical University, Guangzhou, Guangdong, China

**Keywords:** esophageal cancer, home enteral nutrition, experiences, needs, qualitative research

## Abstract

**Objective:**

The benefits of home enteral nutrition for postoperative esophageal cancer patients are well recognized, but there is limited evidence on the experiences and needs of patients and caregivers, particularly in the Chinese context. This study aimed to explore these experiences and identify areas for improvement.

**Methods:**

A descriptive qualitative design with purposive sampling was employed. In-depth, face-to-face interviews were conducted with 13 patients and 15 caregivers at a tertiary hospital in Guangdong Province, China, between November 2024 and January 2025. Thematic analysis was performed using NVivo 14.0 software to organize and interpret the data.

**Results:**

Four main themes emerged from the interviews: Experience-related themes included (1) Physical burden, encompassing tube-related discomfort, gastrointestinal symptoms, restricted activity, sleep disturbances, and fatigue; and (2) Psychological dilemma, including sense of insecurity, stigma, and negative emotions. Needs-related themes included (3) Needs for nutritional management knowledge, such as scientific understanding, knowledge of technical skills, nutritional calculation and weight management, and knowledge for dietary transition; and (4) Needs for external social support, including family support, peer support, and continuity of care support, and social policy support.

**Conclusion:**

This study emphasizes the physical and psychological challenges faced by patients and caregivers during home enteral nutrition, along with their unmet needs for nutritional management knowledge and external support. Healthcare professionals should provide comprehensive information to assist patient decision-making, discharge planning, and nutritional counseling. Improving patients’ and their caregivers’ nutritional literacy and self-care, and utilizing digital platforms and community resources, are essential for enhancing continuity-of-care services and external support systems.

## Introduction

In 2022, esophageal cancer accounted for approximately 511,000 new cases globally, with China contributing nearly half of this disease burden (1). The primary treatment modality for esophageal cancer involves surgical resection, typically combined with adjuvant radiotherapy and chemotherapy. Following esophagectomy, patients undergo a critical transitional period of approximately 30 days, progressing from complete fasting to resuming normal oral intake. With the widespread adoption of enhanced recovery after surgery (ERAS) protocols, the optimal hospitalization duration for esophageal cancer patients has been significantly reduced to 12–13 days (2). However, this abbreviated hospital stay often results in insufficient nutritional recovery, as more than 50% of patients are discharged before achieving adequate oral intake (3). Consequently, postoperative nutritional demands remain a pressing concern, and many patients continue to experience suboptimal nutritional support following discharge.

Home enteral nutrition (HEN) is defined as a method of nutritional support administered at home under medical supervision for clinically stable patients requiring enteral nutrition (4). The primary modalities include enteral tube feeding (ETF) and oral nutritional supplements (ONS) (4). For esophageal cancer patients, the main ETF routes are nasoduodenal and jejunostomy tubes, although the optimal approach remains uncertain (5). While HEN has demonstrated efficacy in providing nutritional support for postoperative esophageal cancer patients (6), evidence regarding patient experiences remains limited and largely non-specific. Patients undergoing HEN often encounter multiple challenges and unmet needs, particularly in physical, psychological, social, and informational domains. Common physical complications include gastrointestinal symptoms, fatigue, and sleep disturbances (7, 8). Beyond these physiological issues, patients frequently report emotional distress, including anxiety, irritability, and depression (9, 10). Moreover, some patients may engage in active or passive social withdrawal due to changes in body image and the impacts of treatment (7, 11). Their need for clear information and reliable healthcare system support is also substantial (12, 13). Alberda et al. (10) emphasized the ongoing requirement for medical guidance, nutritional counseling, and emotional support for both patients and caregivers. Previous qualitative research has further highlighted the need for improved access to routine and emergency community health services, along with reduced financial burdens (14). Comprehensive understanding of these multidimensional challenges could enable healthcare providers to deliver more effective, patient-centered HEN care.

Previous quantitative studies have investigated the efficacy of home enteral nutrition in postoperative esophageal cancer patients, while also documenting associated adverse effects (6, 15, 16). Although qualitative research has examined patients’ perspectives on HEN experiences, the majority of such studies have predominantly focused on long-term HEN recipients (12, 14). Notably, evidence remains particularly scarce regarding the transitional application of HEN following esophageal cancer surgery. Moreover, most existing studies have been conducted in developed countries (9, 14, 17, 18), and their findings may not be fully applicable to the cultural and healthcare contexts of developing countries, particularly in China, where healthcare resources are limited and the caregiving burden is relatively high. Additionally, the perspectives of caregivers who play a crucial role in the implementation of home enteral nutrition have been largely overlooked. Given these gaps, this qualitative study aimed to explore the experiences and specific needs of postoperative esophageal cancer patients and their caregivers during the course of home enteral nutrition in China.

## Methods

This qualitative study was conducted at a tertiary hospital in Guangdong Province, China. The researchers explained the details of the study to the participants and obtained written consent from all participants who were willing to take part in the research. This study received ethical approval from the hospital’s ethics committee (No. NFEC-2024-517).

### Study design

The rationale for conducting this study stems from the lead researcher’s (LHY) dual role as both a nurse and clinical researcher in the Department of Thoracic Surgery, which provided the opportunity for in-depth clinical observations and insights into the topic. Additionally, other members of the research team have been engaged in the study of cancer patients and their care needs within their respective areas of expertise. Accordingly, a descriptive qualitative design based on thematic analysis was adopted for this study. Descriptive qualitative research is intended to explore participants’ thoughts, perceptions, and experiences related to a particular phenomenon (19). Given this objective, we considered a descriptive qualitative approach appropriate to explore esophageal cancer patients’ and their caregivers’ perspectives, experiences, expectations, and needs regarding home enteral nutrition following surgery.

### Sample and setting

This study was conducted between November 2024 and January 2025 at a tertiary care hospital in Guangdong Province, China. Inclusion criteria for patients were as follows: individuals who had undergone esophagectomy and were receiving home enteral nutrition; had regular follow-up and treatment in the thoracic surgery department; were aged 18 years or older; demonstrated adequate communication abilities; voluntarily agreed to participate; and provided written informed consent. Exclusion criteria for patients included: presence of severe complications, cardiovascular or cerebrovascular disease, other malignancies, significant cognitive impairment or psychiatric disorders, receipt of palliative care, or lack of awareness of their diagnosis. Caregivers were eligible if they were the primary caregiver of the patient and were willing and able to express their experiences and perspectives clearly. Caregivers were excluded if they had notable communication or comprehension difficulties. To ensure a diverse range of experiences, patients and caregivers were purposively selected to reflect variability in age, pathological stage, financial situation, as well as the mode and duration of HEN. Participant recruitment continued until no new information or themes emerged from the interviews, indicating that data saturation had been achieved (20).

### Data collection

Face-to-face, semi-structured interviews were conducted by the principal investigators and audio-recorded. Interview sessions were scheduled at times and locations convenient for participants. The interview guide was developed based on a review of relevant literature and the research team’s clinical experience in this field. It was refined after conducting pilot interviews with one patient and one caregiver. The final interview guide is provided in Supplementary material. Data from the two pilot participants were included in the final analysis. Prior to each interview, participants received a detailed explanation of the study objectives and provided written informed consent. The research team ensured that interviews took place in quiet, comfortable settings free from potential disruptions. Patients and caregivers were interviewed separately. Field notes were taken during the interviews, and clarifications were sought from participants when necessary. No repeat interviews were conducted. Interviews were concluded when participants had no further information to share. Each interview lasted approximately 20 to 60 min and was audio-recorded in its entirety.

### Data analysis

All interviews were audio-recorded and transcribed verbatim within 48 h of completion. A Chinese speech-to-text software, FeiShu, was used for the initial transcription, which was then carefully reviewed and cross-checked multiple times by the first to fourth authors to ensure accuracy and credibility. For participants with speech impairments or those who spoke in regional dialects, manual transcription was performed.

The transcripts were imported into NVivo 14.0 software for textual analysis to identify key issues and themes relevant to the research objectives (21). The thematic analysis followed these steps: (i) two authors (LHY and CMX) read the transcripts repeatedly to become familiar with the data; (ii) the same authors independently developed and generated initial codes; (iii) relevant data segments were collated under each code, which were then grouped into potential themes; (iv) the themes were reviewed through iterative discussions among the authors; (v) themes were defined and named; and (vi) the final report was produced. In cases of disagreement, the researchers revisited the original transcripts and consulted with another author (LM) until consensus was reached.

### Ethical considerations

Throughout the research process, careful measures were taken to ensure informed consent and to protect participants’ privacy and confidentiality. Patients were explicitly informed that participation was voluntary and that they could withdraw from the study at any time without affecting their subsequent medical care. To prevent the disclosure of personal information, all transcribed data were anonymized. Each participant was assigned a unique identification code (e.g., Patient 1 as P1, Caregiver 1 as C1). Consent forms and audio recordings were securely stored in the private office of the lead researcher, in a locked cabinet accessible only to the research team.

## Results

### Demographics of participants

Tables 1, 2 present the characteristics of the study participants. A total of 13 patients and 15 caregivers were invited to participate in this study. The patients ranged in age from 52 to 70 years, with a mean age of 61.2 years. The majority had a monthly income at a mid-to-low level. The caregivers’ ages ranged from 21 to 70 years, with a mean age of 46.6 years, and were primarily the spouses or children of the patients.

**Table 1:** Demographic and clinical characteristics of patients.

ID	Gender	Age (years)	Pathological stage	Monthly household income per capita (Chinese yuan)	HEN modality	Duration of HEN (days)
P1	Male	52	IB	3,000–5,000	Nasoduodenal	7
P2	Male	62	IVA	3,000-5,000	Nasoduodenal	34
P3	Female	57	IIB	3,000-5,000	Jejunostomy	19
P4	Male	62	IIIA	3,000-5,000	Jejunostomy	31
P5	Male	63	IIB	≥10,000	Nasoduodenal	23
P6	Male	58	IIIA	3,000-5,000	Nasoduodenal	32
P7	Male	70	IB	5,000-10,000	Nasoduodenal	14
P8	Male	60	IIIA	5,000-10,000	Jejunostomy	29
P9	Male	63	IIB	5,000-10,000	Jejunostomy	43
P10	Male	68	IIIA	3,000-5,000	Nasoduodenal	91
P11	Male	54	IA	5,000-10,000	ONS & Jejunostomy	36
P12	Female	64	IIB	3,000-5,000	Nasoduodenal	15
P13	Male	63	IIIA	3,000-5,000	ONS and Jejunostomy	32

**Table 2:** Demographic characteristics of caregivers.

ID	Age (years)	Relationship to patient	Monthly household income per capita (Chinese yuan)	Patient’s HEN modality	Duration of patient’s HEN (days)
C1	60	Spouse	3,000-5,000	Nasoduodenal	12
C2	52	Spouse	3,000-5,000	Nasoduodenal	7
C3	60	Spouse	3,000-5,000	Nasoduodenal	34
C4	40	Adult children	≥10,000	ONS and Nasoduodenal	70
C5	63	Spouse	3,000-5,000	ONS and Nasoduodenal	49
C6	60	Spouse	3,000-5,000	Nasoduodenal	53
C7	53	Spouse	3,000-5,000	Jejunostomy	31
C8	35	Adult children	≥10,000	Nasoduodenal	23
C9	28	Adult children	3,000-5,000	Nasoduodenal	32
C10	70	Spouse	5,000-10,000	Nasoduodenal	14
C11	21	Adult children	5,000-10,000	Jejunostomy	29
C12	35	Adult children	5,000-10,000	Jejunostomy	10
C13	27	Daughter in-law	5,000-10,000	Jejunostomy	43
C14	40	Adult children	3,000-5,000	Nasoduodenal	91
C15	55	Spouse	5,000-10,000	ONS and Jejunostomy	36

### Theme 1: Physical burden

#### Subtheme 1: tube-related discomfort

Patients receiving home enteral nutrition after esophagectomy commonly reported discomfort associated with feeding tubes, particularly among those using nasoduodenal tubes. In this study, nearly all patients receiving nasogastric feeding or their caregivers reported tube-related discomfort. These patients frequently described sensations such as throat irritation, foreign body sensation, and abdominal pulling. Skin discomfort was also prevalent, often due to prolonged use of adhesive tape and pressure-induced injuries around the nasal area. This physical discomfort sometimes led to psychological responses such as fear of movement, resulting in reduced daily activity.

*“He always feels that the tape on his nose is itchy, and often cannot help but touch the area with his hand. He keeps complaining about pain where the tube goes through his throat. He says there’s a foreign body sensation, and the skin around his nostrils has broken due to constant friction.”* (C4)

In contrast, patients using jejunostomy tubes reported fewer issues directly related to the tube itself but described discomfort around the stoma site. Six participants, P3, P4, P8, C7, C11, and C12, mentioned pain related to the stoma site. Pain was often attributed to wound complications or tension from sutures.

*“I’m not sure if the wound has not healed yet, but it hurts when the nutrition solution leaks onto the site. And the stitches holding the tube feel too tight. The wound feels like it’s under a lot of tension, and sometimes it just aches.”* (P4)

#### Subtheme 2: gastrointestinal symptoms

Nearly all participants reported experiencing gastrointestinal symptoms during the HEN period, often due to poor tolerance of enteral formulas or inappropriate infusion speed. Reported issues included diarrhea, bloating, constipation, and reflux, all of which negatively influenced patients’ daily functioning and quality of life. Several participants described experiencing frequent, watery stools or alternating bowel irregularities, which were distressing and raised concerns about potential complications.

*“There was a period when I had diarrhea four or five times a day. Every time the formula was infused, it caused a bloated feeling. My stomach did not hurt, but eventually the frequent diarrhea led to bleeding.”* (P1)

*“Sometimes I went three or four days without a bowel movement even though I was getting so much nutrition every day. I wasn’t sure if that was normal.”* (P2)

Oral nutritional supplements triggered nausea and vomiting in some patients due to their unpleasant taste. As one participant described, *“I vomited both times I tried it. Just the smell made me nauseous.”* (P13).

#### Subtheme 3: restricted activity

Seven patients, P1, P3, P5, P7, P9, P11, and P13, reported experiencing activity restrictions during home enteral nutrition due to various factors, which negatively impacted their daily lives. Patients reported that the prolonged duration of enteral nutrition infusions, particularly when delivered via feeding pumps, imposed various limitations on their physical activity and daily routines. Continuous or long-hour feeding often confined patients to a fixed space, reducing their opportunities for mobility and social engagement. In addition to mobility constraints, the presence of feeding tubes also caused inconvenience in personal hygiene, such as washing, shaving, or bathing. Environmental considerations, such as avoiding high temperatures to prevent formula spoilage, further restricted patients from going outdoors. These activity limitations collectively contributed to decreased autonomy and a reduced quality of life during the HEN period.

*“Back then, I was receiving nutrition around the clock. I had to stay in bed all day and did not have a chance to go outside or move around.”* (P3)

*“We hardly went outside because of the sun. The formula cannot be exposed to heat, so we had to stay indoors most of the time.”* (P5)

#### Subtheme 4: sleep disturbances

Sleep disturbances were commonly reported among patients undergoing home enteral nutrition, especially when nighttime feeding was required. Postoperative esophageal cancer patients are often instructed to maintain specific sleeping postures, such as a semi-upright position, to reduce reflux and aspiration risks. However, this unnatural positioning significantly affected sleep comfort. In addition, the need to remain relatively still to avoid tube dislodgement, as well as frequent interruptions due to formula administration or pump alarms, further compounded sleep difficulties. As a result, patients often experienced fragmented sleep, limited to a few hours at a time, which negatively impacted their daytime functioning and overall well-being.

*“He has to sleep with a cushion at a 30- to 40-degree angle, which feels very unnatural. Plus, since the nutrition is being infused while he sleeps, he cannot move around. He has to stay in one position to avoid pulling the tube, and it’s very uncomfortable. Sometimes he only sleeps three or four hours and feels exhausted during the day.”* (C12)

*“When I sleep at night, if I roll over and put pressure on the tube, it becomes uncomfortable. Then the formula finishes in about an hour, and I have to wake up again to change it. And the alarm on the machine is really loud and piercing. I have to get up frequently to turn it off.”* (P5)

#### Subtheme 5: fatigue

Fatigue emerged as a prevalent concern among patients receiving home enteral nutrition following esophagectomy. The combined effects of major surgical trauma, suboptimal nutritional status, and the ongoing physiological burdens associated with prolonged tube feeding contributed to diminished physical energy and endurance. In this subtheme, P2, P3, P4, P8, and P9 mentioned the impact of weakness and fatigue on their lives since starting home enteral nutrition.

*“When I come back to the hospital for check-ups, I cannot even stand properly while waiting. I have to sit down until my number is called. If no one is with me, I’d probably need a cane. It’s like getting a preview of old age.”* (P3)

*“I just do not have the energy. I can walk, but doing anything else is too tiring. Even taking a shower wears me out. I have to sit while bathing now. I used to grow vegetables, but now the field is overgrown. Everything I do feels slow and weak.”* (P9)

### Theme 2: Psychological dilemma

#### Subtheme 1: sense of insecurity

The shift from hospital to home enteral nutrition care caused significant insecurity for patients and caregivers. Transferring responsibility to untrained individuals led to anxiety, fear of errors, and low confidence. Without real-time professional support, many felt vulnerable, and some initially resisted home enteral nutrition due to fear and lack of preparedness.

*“Home is not like the hospital. You’re all so professional there. At home, it felt like we suddenly became the nurses. We’re on our own, and I’m constantly worried that if something feels wrong, maybe it’s because I did something incorrectly.”* (P4)

*“We thought all the tubes would be removed before discharge. We had never dealt with anything like this. Then suddenly at home we had to do everything ourselves. It was chaotic and terrifying.”* (P5)

#### Subtheme 2: stigma

The study identified notable heterogeneity in the experience of illness-related shame or embarrassment among patients receiving home enteral nutrition, which appeared closely related to the type of feeding modality used. In this study, nearly all patients receiving nasogastric feeding reported feeling embarrassed and ashamed due to this feeding method. Patients using nasoduodenal tubes, which are visibly positioned on the face and extend through the nasal passage, reported a stronger sense of embarrassment and body image disturbance. This visibility often triggered self-consciousness, discomfort in social settings, and a tendency to avoid public outings altogether. The perception of being visibly different or attracting attention due to the tube placement created psychological barriers that suppressed patients’ motivation to engage in normal social activities.

*“Even with a mask on, the tube is still visible—people stare, and I feel ashamed; I do not even dare go out with this tube on my face, looking pale and awful.”* (P10).

In contrast, patients using jejunostomy tubes, which are concealed under clothing and less externally apparent, expressed fewer concerns about public visibility. These patients were more likely to maintain outdoor activities and social participation when physically capable. The relative discretion of the jejunostomy tube seemed to preserve their sense of normalcy, allowing them to reengage with social life to some extent.

*“Since the tube is hidden under clothes, I can go out looking normal if I have the strength, which is why we chose the abdominal placement in the first place.”*(P11).

#### Subtheme 3: negative emotions

During the course of home enteral nutrition, many patients reported experiencing a range of negative psychological states, including irritability, emotional withdrawal, sadness, low self-esteem, and in more severe cases, depressive symptoms. These emotional disturbances were especially evident among individuals with prolonged HEN durations or those who developed postoperative complications such as anastomotic leaks or strictures. The prolonged physical discomfort, combined with the ongoing dependence on medical devices and the perceived stagnation of recovery, contributed to a sense of helplessness and psychological burden.

*“Later, he had a stent placed due to esophageal stricture, which meant he had to continue on enteral feeding. His mood became very unstable. Sometimes, when I was helping with the feeding, he would suddenly lash out at me for no reason.”* (C15)

*“Then his throat wound started leaking. We had to use both enteral and parenteral nutrition again. It was harder than the surgery or hospitalization. My husband said during that time, he even thought about jumping off a building.”* (C5)

### Theme 3: Needs for nutritional management knowledge

#### Subtheme 1: scientific understanding

Some patients and caregivers lacked understanding of the necessity of continued home enteral nutrition after esophagectomy, often due to misconceptions about recovery and insufficient discharge education. In this subtheme, nine participants, P1, P2, P6, P9, P10, C2, C10, C12, and C13, expressed doubts about the necessity and importance of home enteral nutrition after surgery. Many viewed the feeding tube as a temporary hospital-only measure, leading to confusion or resistance when told it must continue at home. They questioned the adequacy of tube feeding, worried about its impact on normal eating, and often misattributed symptoms like dysphagia or weight loss to the tube itself.

*“He keeps saying nonsense. He thinks the surgeons sewed his throat too tight during the operation, and that’s why they inserted the tube and will not let him eat. By the way, he has not eaten any real food and just keeps getting nutrition through the tube. Is that really enough? Will it affect his ability to eat normally in the future?”* (C13)

*“When I was in the hospital, the patient in the bed next to me was discharged without a tube. I assumed I’d be able to eat too once I left, so I do not understand why I have to go home with a feeding tube.”* (P1)

*“I actually lost weight while using the feeding tube. Does it really provide nutrition? But this was something you made mandatory, so I did not have a choice.”* (P2)

#### Subtheme 2: knowledge of technical skills

Many patients and caregivers, despite receiving basic HEN training before discharge, struggled with home implementation due to limited troubleshooting skills. Uncertainty about flushing, tube care, formula storage, and equipment use led to anxiety, errors, and safety risks, highlighting the need for ongoing practical support.

*“I always feel like I’m doing the tube flushing wrong, there’s always something stuck inside, and over time it seems like a lot of residue builds up. Also, sometimes the formula milk smells a little sour after being stored for a while. Could it be spoiled? I’m really not very professional, and there were times I pressed too hard, causing milk to spray everywhere and get all over him. And the machine keeps making a noise; I do not know if it’s a problem with the machine itself or if there’s an issue with the tube. I just cannot figure it out.”* (C4)

#### Subtheme 3: nutritional calculations and weight management

Postoperative esophageal cancer patients often have poor nutritional status, which can worsen during HEN due to feeding-related complications, hindering recovery. C4, C8, C9, C12, and C13, who are younger and more educated caregivers, expressed a strong need to master nutritional calculation and management. Younger caregivers, in particular, expressed a need for guidance on calculating caloric requirements, monitoring intake, and managing weight. They were confused about fluctuating energy needs and adjusting formulas accordingly, and worried about the risks of underfeeding or overfeeding affecting recovery.

*“The doctor said he needs over 2,100 calories a day, but even if we finish a whole can of formula, it only adds up to a little over 1,500. Isn’t that too little? Or have his needs changed? I want to understand how this is calculated so we can make adjustments ourselves.”* (C9)

*“When we were discharged, we followed the nurse’s instructions to adjust the powder, but he kept losing weight. Later, we tried the doctor’s recommended dosage for a month and he gained about 11 pounds, maybe it is too fast. I really want to know how to find the right balance.”* (C4)

#### Subtheme 4: knowledge of dietary transition

Following esophagectomy, patients on HEN must gradually progress through a complex dietary transition—from liquids to semi-liquids, soft foods, and eventually a normal diet. Swallowing difficulties were frequently cited as a significant barrier, impeding this transition. Despite the clinical importance of dietary advancement, most participants reported lacking practical guidance regarding the timing of each phase, appropriate food textures, and the need for additional nutritional supplementation. Many felt uncertain about what was safe to consume and how to interpret informal advice.

*“Since my esophagus became narrow and tight after the surgery, swallowing is difficult, and I’m unsure about what I can eat, but a friend suggested I should eat more solid foods to help stretch it out instead of sticking to soft foods—I’m not sure if that’s true.”* (P12).

Some patients expressed a desire for culturally adapted dietary guidance. They hoped to enhance their nutrition through traditional food-based remedies and sought advice aligned with local Chinese dietary customs.

*“Would it be okay for me to have some Chaoshan-style fish maw congee (a gelatinous preparation derived from fish swim bladder)? I’m just not sure if it’s easy to digest.”* (P11).

*“My family is thinking of making a five-finger fig root herbal decoction (a TCM-nourishing soup). Do you think that would be suitable for my nutritional needs?”* (P2)

### Theme 4: Needs for external social support

#### Subtheme 1: family support

As key participants in the implementation of home enteral nutrition, family members play a vital role in supporting patients throughout the process. Most patients reported receiving significant support from their families during the HEN period. Such involvement not only helped reduce caregiving burdens but also provided essential emotional comfort and motivation.

*“My wife kept telling me to hang in there and that everything would be better once the tube came out. After having the tube removed and being discharged from the hospital, I was in such a good mood. My cousin picked me up right away and brought me to his place. I had a bowl of soft porridge there, and honestly, it tasted even better than bird’s nest soup.” (laughs)* (P3)

*“The day he had his tube removed, my dad called me on video. He was smiling—really smiling—for the first time since his surgery. He looked so happy, like a little kid.” (tearing up)* (C14)

Apart from emotional support, family caregivers were also responsible for providing high-quality HEN care. Some caregivers had to adapt quickly and take on new responsibilities, which could be particularly challenging.

*“Caring for me has been really hard on my wife. I used to be the one looking after her. Now she had to learn everything from scratch, such as how to mix the formula and how to administer the nutrition. She also had to stay close to me all the time. It’s really been a lot for her.”* (P5)

#### Subtheme 2: peer support

In this subtheme, seven participants, P6, P9, P12, C4, C9, C12, and C14, emphasized the positive role of peer support in facilitating the exchange of nutritional resources. Peer support, alongside professional and familial support, provided meaningful benefits for patients and caregivers. Participants found comfort in connecting with others who had similar experiences, sharing caregiving strategies, resources, and emotional support, which helped normalize challenges, reduce isolation, and improve coping.

*“When I came for a follow-up visit, I saw another patient using a portable infusion pump, and I thought that was really convenient. So I asked them for the purchase link. Honestly, when you have been through something like this, sharing information like that is really helpful.”* (C14)

*“Sometimes we cannot finish all the formula at home, so we post the extras in the patient Wechat group to sell or give away.”* (C9)

#### Subtheme 3: continuity of care support

Thirteen participants, P4, P6, P8, P9, P11, P13, C2, C4, C8, C9, C11, C12, C13, stressed the need for ongoing professional support after hospital discharge. Despite HEN training, they struggled with managing procedures at home, especially during complications. They desired continued remote guidance via phone or digital platforms like WeChat, along with in-person follow-ups, to build confidence, ensure safety, and reduce post-discharge anxiety.

*“I think the hospital’s follow-up system and communication channels are really inconvenient, especially for patients like us who live far away. When something goes wrong, there’s no clear way to contact the primary physician. Every time we run into a problem, we feel helpless. It’s frustrating and stressful. Honestly, if someone could have come to the house to teach us during the early stages, that would’ve made a huge difference.”* (C4)

*“One time, the feeding tube got clogged while we were administering medication. We rushed to the hospital, only to find out that we could have cleared it using baking soda. If someone had just messaged us on WeChat, we would not have had to make the trip.”* (P9)

Beyond hospital care, some participants advocated for enhanced support from local community healthcare providers, particularly in delivering services compatible with HEN requirements.

*“I wanted to go to the community clinic for help with administering the nutrition, but they did not have a pump that worked with our tubing setup, so I gave up.”* (P6)

#### Subtheme 4: social policy support

The financial burden associated with home enteral nutrition emerged as a prominent concern among both patients and caregivers. In this subtheme, 12 participants, P2, P3, P6, P8, P10, P11, C3, C4, C6, C7, C9, and C14, who had lower monthly incomes and a longer duration of home enteral nutrition, highlighted the considerable financial burden that resulted from the cost of nutritional care. Despite its clinical necessity, the cost of nutritional products and related equipment is not currently covered under China’s mainstream medical insurance policies. This gap in reimbursement policies has placed substantial pressure on many families, especially those with limited income or lacking comprehensive insurance coverage at the time of treatment.

*“I was under a lot of pressure. From the time I had surgery until about two months later, I did not have any insurance coverage, so every single bottle of enteral nutrition formula came directly from our savings. The most substantial out-of-pocket expenditure throughout our treatment journey has undoubtedly been nutritional support. I’d estimate it accounts for over half our total medical costs.”* (P3)

To save costs, many families resorted to purchasing enteral nutrition products online, although this came with added concerns about quality and safety.

*“We bought the formulas on Taobao (China’s predominant e-commerce platform), but I was always worried whether the products were up to standard since they did not come from the hospital. At one point, we looked into buying a feeding pump, but it cost around 2,000 yuan online. It just felt too expensive, so we did not get it.”* (C2)

## Discussion

This study provides new evidence on the experiences and needs of postoperative esophageal cancer patients receiving home enteral nutrition within the Chinese cultural context. By including both patients and family caregivers, the study captured perspectives that patients may have been unwilling or unable to express themselves, thereby enriching the depth and breadth of the findings. The results highlight patients’ specific needs for nutritional management knowledge, as well as their multidimensional needs for external support. Among these, accessibility to medical resources and HEN-related services was frequently emphasized. In contrast, support for continuity of care was commonly perceived as insufficient, indicating room for improvement in this area.

Although home enteral nutrition is essential for the survival of postoperative esophageal cancer patients, it is often associated with a range of physical discomforts. Similar to findings from previous studies, patients in the current study reported common physiological issues, including gastrointestinal symptoms, restricted mobility, sleep disturbances, and fatigue (7, 9, 22). Tube-related physical discomfort, which has received limited attention in earlier research, was frequently mentioned by participants. These discomforts were mostly caused by the insertion of feeding tubes. Patients with nasoduodenal tubes often experienced a sensation of a foreign body in the throat and intestinal traction, while those with jejunostomy tubes mainly reported wound pain caused by sutures or infections. In China, decisions regarding HEN modalities are typically made by healthcare providers with limited involvement from patients themselves (23). This highlights the importance of informing patients about possible sources of discomfort and considering their preferences when selecting feeding methods. Doing so may help improve patients’ physical comfort and treatment adherence. Gastrointestinal discomfort is one of the most common complications of HEN. In this study, constipation and abdominal bloating were frequently reported, which is consistent with previous findings (7). Previous research has shown that skills training prior to hospital discharge significantly reduces gastrointestinal symptoms 3 months after discharge (24), underscoring the importance of standardized discharge education for patients and caregivers. In addition to gastrointestinal symptoms, participants also reported physical limitations and sleep disruptions, which are consistent with findings in earlier studies (7, 9, 22). Fatigue was commonly observed and may be attributed to the combined effects of physical discomfort, poor sleep quality, and inadequate nutrition (25). Healthcare providers are encouraged to guide patients in engaging in home-based physical activities that may help improve sleep and reduce fatigue. Activities such as Baduanjin, yoga, and dancing have shown potential benefits in this regard (26–28). The accumulation of physical discomfort during the HEN period can significantly compromise the quality of life for patients (29, 30). Therefore, healthcare professionals should prioritize interventions that address these issues and offer practical guidance to relieve physical burden and improve overall well-being.

Consistent with previous findings, patients in this study described a range of psychological dilemmas during the period of HEN, encompassing feelings of insecurity, illness-related stigma, and various negative emotional states. Participants commonly reported a sense of insecurity stemming from uncertainty about the future and inadequate preparation for home care, which can hinder caregiving and reduce quality of life (31). Prior research has identified inadequate discharge planning (32) and ineffective communication (33), highlighting the need for tailored discharge plans to boost confidence and safety at home. Stigma, especially among patients with nasoduodenal tubes, led to shame and social withdrawal, more so than in those with jejunostomy, aligning with prior studies (7, 11, 34, 35). To reduce stigma, public education should be strengthened, and psychosocial factors considered when selecting feeding methods, with professional support favoring jejunostomy when appropriate. Negative emotions such as anxiety and depression were also common, often linked to prolonged tube feeding and delayed return to oral intake. Since HEN is typically temporary after esophageal surgery (9), healthcare providers should promote a positive mindset and active coping strategies to support recovery.

Access to health information is a common unmet need among cancer patients and caregivers (36), with this study highlighting a specific gap in nutritional knowledge. When such needs are not adequately addressed, patients may experience increased physical and psychological burdens, and even compromised nutritional status (37). Many lacked understanding of home enteral nutrition after esophageal surgery, with some holding misconceptions likely due to limited involvement in HEN-related decisions. This can reduce adherence and disrupt nutritional support (13). Healthcare providers should clearly communicate the necessity and benefits of HEN and reinforce this throughout care. Although most patients and caregivers received HEN education before discharge, many reported unmet needs during implementation, especially in tube care and medication management—echoing past studies (14, 38, 39). Younger, well-educated caregivers often sought advanced knowledge like weight control and nutrient calculation, aiming to “become experts” in care. This highlights the importance of tailoring educational support to this population to promote self-management and facilitate the progress of HEN. Moreover, since HEN is a temporary nutritional support strategy for postoperative esophageal cancer patients (9), eventual transition to oral feeding is inevitable. Both patients and caregivers expressed a strong need for knowledge regarding this dietary transition phase. Notably, since Guangdong Province is one of the high-incidence areas for esophageal cancer in China, the nutritional knowledge needs of patients in this region also reflect a fusion of regional cultural characteristics and traditional Chinese medicine (TCM) beliefs. In TCM, food and medicine share the same origin (40), and many patients hoped to supplement their nutritional intake not only through commercial formulas, but also through culturally meaningful foods believed to replenish “qi and blood” which closely related to nutrition and internal balance. This suggests that healthcare professionals should take patients’ cultural backgrounds into account when offering dietary transition guidance, providing individualized advice. It also underscores the need for a broader and deeper knowledge base among healthcare providers.

Enhancing social support is vital for patients managing home enteral nutrition. Caregivers, who shoulder key responsibilities post-esophagectomy, often lack adequate knowledge, as noted in this study and by Xue (12). This suggests the need for dual-targeted educational programs and comprehensive support for both patients and caregivers. Similar to Alberda’s study (10), participants emphasized the positive impact of peer support. Healthcare providers could facilitate the formation of online support groups, such as WeChat groups, to encourage shared resources and emotional connection. In addition, a strong demand for multi-channel continuity-of-care support was identified. Follow-up via phone and WeChat has been shown to improve nutrition and quality of life in post-esophagectomy patients (41). Therefore, implementing regular, multi-modal follow-up interventions may yield significant benefits. Moreover, Patients also raised concerns about limited community resources. The “hospital-community-family” model, effective in chronic disease management (42–44), may benefit HEN patients by strengthening community healthcare as a bridge between hospital and home. Economic burden was a common concern, with participants calling for national subsidies and standardized channels for purchasing HEN supplies. Currently, China lacks centralized systems or financial support for discharged patients. National standards are urgently needed to streamline HEN management, ease costs, and improve access (17).

### Strengths and limitations of study

This is the first known study in China to explore the experiences and needs of postoperative esophageal cancer patients on home enteral nutrition from both patient and caregiver perspectives, which may facilitate the future clinical supportive care. However, it has limitations. Conducted in a single tertiary hospital in Guangdong, the findings may not be widely generalizable. Voluntary participation may have introduced selection bias, excluding those too ill or hoarse to join. Additionally, varied feeding methods introduced heterogeneity, particularly in discomfort and stigma, though this diversity also enriched the insights and supported patient-centered decision-making.

### Future research direction

Based on the findings of this study, several aspects can be explored in future research to enhance our understanding and support of postoperative esophageal cancer patients receiving home enteral nutrition. First, longitudinal follow-up studies could be conducted to track changes in nutritional status, quality of life related to home enteral nutrition, and both physical and psychological conditions throughout the process. This would provide insights into the evolving nutritional needs of patients. Additionally, future studies could investigate the real-life experiences and quality of life of patients using different nutritional support methods, particularly those receiving nutrition through nasoduodenal and jejunostomy tubes, to better understand the specificity of their nutritional needs and provide evidence for future best practices and clinical decision-making. Finally, intervention-based studies are needed to develop nutritional support programs that address both patient and caregiver needs, with targeted nutritional education and robust external support. These efforts are crucial for improving the quality of life of esophageal cancer patients during the home enteral nutrition process.

## Conclusion

Esophageal cancer patients undergoing home enteral nutrition face multiple physical, psychological, and social challenges. This study underscores the urgent need to enhance self-management skills and strengthen external support systems. Participants described various obstacles and coping strategies during HEN. Healthcare professionals should provide effective dual-targeted guidance to relieve gastrointestinal symptoms, reduce discomfort, insecurity, and stigma, and promote active coping to boost recovery confidence. Particular attention should be given to the supportive care needs of both patients and caregivers, including nutritional self-management, material support, and continuity of care throughout the HEN period.

## Data Availability

The original contributions presented in the study are included in the article/Supplementary material, further inquiries can be directed to the corresponding author.
