# Supplementary material for: Focusing on the process: experiences and needs of Chinese post-esophagectomy patients and caregivers regarding home enteral nutrition
Source: Front Nutr. 2025 Jul 24;12:1636203. doi: 10.3389/fnut.2025.1636203 (PMC12328186; doi:10.3389/fnut.2025.1636203)
Supplement: Supplementary file 1 [file Table_1.DOCX]

Supplementary Material

# Questions on the Semi-Structured Interview Form

For Patients:

1.Could you please describe your experiences and feelings during the process of receiving Home Enteral Nutrition?

2.In what ways has HEN impacted your daily life?Can you give some examples?

3.What kinds of difficulties or barriers have you encountered while undergoing HEN? How did you address or manage them?

4.What concerns or worries have you had during this process?

5.What kind of support or needs would you hope to receive during the implementation of HEN?

For Family Caregivers:

1.How have you felt while caring for the patient during their Home Enteral Nutrition?

2.What kinds of difficulties or barriers have you faced in the caregiving process? How did you cope with them?

3.What concerns or worries have you experienced throughout this process?

4.What kinds of support or needs would you hope to receive as a caregiver?
